# The genome sequence of the Lesser Broad-bordered Yellow Underwing, *Noctua janthe* (Borkhausen, 1792)

**DOI:** 10.12688/wellcomeopenres.19412.1

**Published:** 2023-04-27

**Authors:** Douglas Boyes, Peter W.H. Holland, University of Oxford and Wytham Woods Genome Acquisition Lab, Darwin Tree of Life Barcoding collective, Wellcome Sanger Institute Tree of Life programme, Wellcome Sanger Institute Scientific Operations: DNA Pipelines collective, Tree of Life Core Informatics collective, Darwin Tree of Life Consortium

**Affiliations:** 1 UK Centre for Ecology & Hydrology, Wallingford, England, UK; 2 University of Oxford, Oxford, England, UK

**Keywords:** Noctua janthe, Lesser Broad-bordered Yellow Underwing, genome sequence, chromosomal, Lepidoptera

## Abstract

We present a genome assembly from an individual male *Noctua janthe* (the Lesser Broad-bordered Yellow Underwing; Arthropoda; Insecta; Lepidoptera; Noctuidae). The genome sequence is 532.8 megabases in span. Most of the assembly is scaffolded into 31 chromosomal pseudomolecules, including the Z sex chromosome. The mitochondrial genome has also been assembled and is 15.3 kilobases in length. Gene annotation of this assembly on Ensembl identified 17,653 protein coding genes.

## Species taxonomy

Eukaryota; Metazoa; Ecdysozoa; Arthropoda; Hexapoda; Insecta; Pterygota; Neoptera; Endopterygota; Lepidoptera; Glossata; Ditrysia; Noctuoidea; Noctuidae; Noctuinae; Noctuini; *Noctua*; *Noctua janthe* (Borkhausen, 1792) (NCBI:txid987995).

## Background

The Lesser Broad-bordered Yellow Underwing, *Noctua janthe*, is a moth in the family Noctuidae found across most of the UK and northern Europe, with additional scattered records from Italy, Spain and Greece ( GBIF Secretariat, 2022; Randle *et al.*, 2019). The moth is common in the southern counties of England and the adults are strongly attracted to light. There has been much taxonomic discussion around whether *N. janthe* is a distinct species from Langmaid’s yellow underwing, *Noctua janthina* ( Townsend *et al.*, 2010). The latter was first recorded in the UK from Southsea, Hampshire, in 2001 ( Langmaid, 2002) and is a rarer, suspected immigrant moth, now resident in small numbers on the south coast of England ( Randle *et al.*, 2019). Both *N. janthe* and *N. janthina* have brown forewings with similar mottled patterns, and yellow-orange hindwings with black markings. The black markings on the hindwing upper side are more extensive in *N. janthina*, particularly in males, and there are subtle but consistent differences in genitalia morphology suggestive of species-level distinction ( Townsend *et al.*, 2010). Phylogenetic analysis of DNA barcode data also suggests that *N. janthe* and *N. janthina* are different species ( P.O. Mulhair’s analysis at Barcode of Life Data Systems, n.d.). There are currently insufficient molecular data to confirm if another very similar European moth, *N. tertia*, is also a distinct species.

*N. janthe* is found in gardens, hedgerows, grasslands and occasionally woodland, where the larvae feed on a wide range of trees and herbaceous plants. In the UK, there is one generation per year, with the adults on the wing in July and August, and the larvae developing in autumn before overwintering then continuing larval development in spring. Many noctuid moths are prey for bats; experiments using *N. janthe* in tethered flight revealed that ultrasonic pulses elicit a sudden increase in flight strength, which is likely an evasive response ( Hügel & Goerlitz, 2019).

A genome sequence of *Noctua janthe* will facilitate understanding of molecular adaptations to polyphagy, and will contribute to a growing data set of resources for understanding lepidopteran biology.

## Genome sequence report

**Figure 1.**
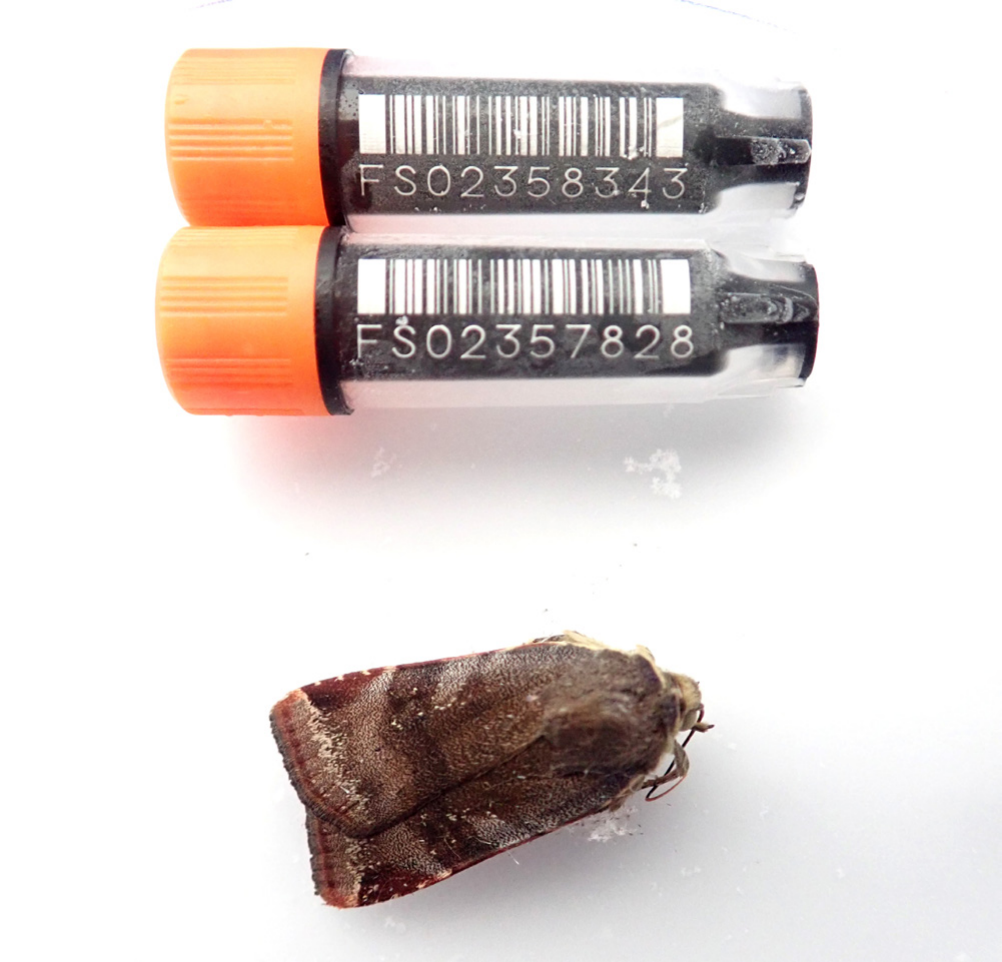
Photograph of the *Noctua janthe* (ilNocJant1) specimen used for genome sequencing.

The genome was sequenced from one male *Noctua janthe* ( Figure 1) collected from Wytham Woods, Oxfordshire, UK (latitude 51.77, longitude –1.34). A total of 48-fold coverage in Pacific Biosciences single-molecule HiFi long reads and 79-fold coverage in 10X Genomics read clouds were generated. Primary assembly contigs were scaffolded with chromosome conformation Hi-C data. Manual assembly curation corrected eight missing joins or mis-joins and removed three haplotypic duplications, reducing the scaffold number by 17.95%.

**Figure 2.**
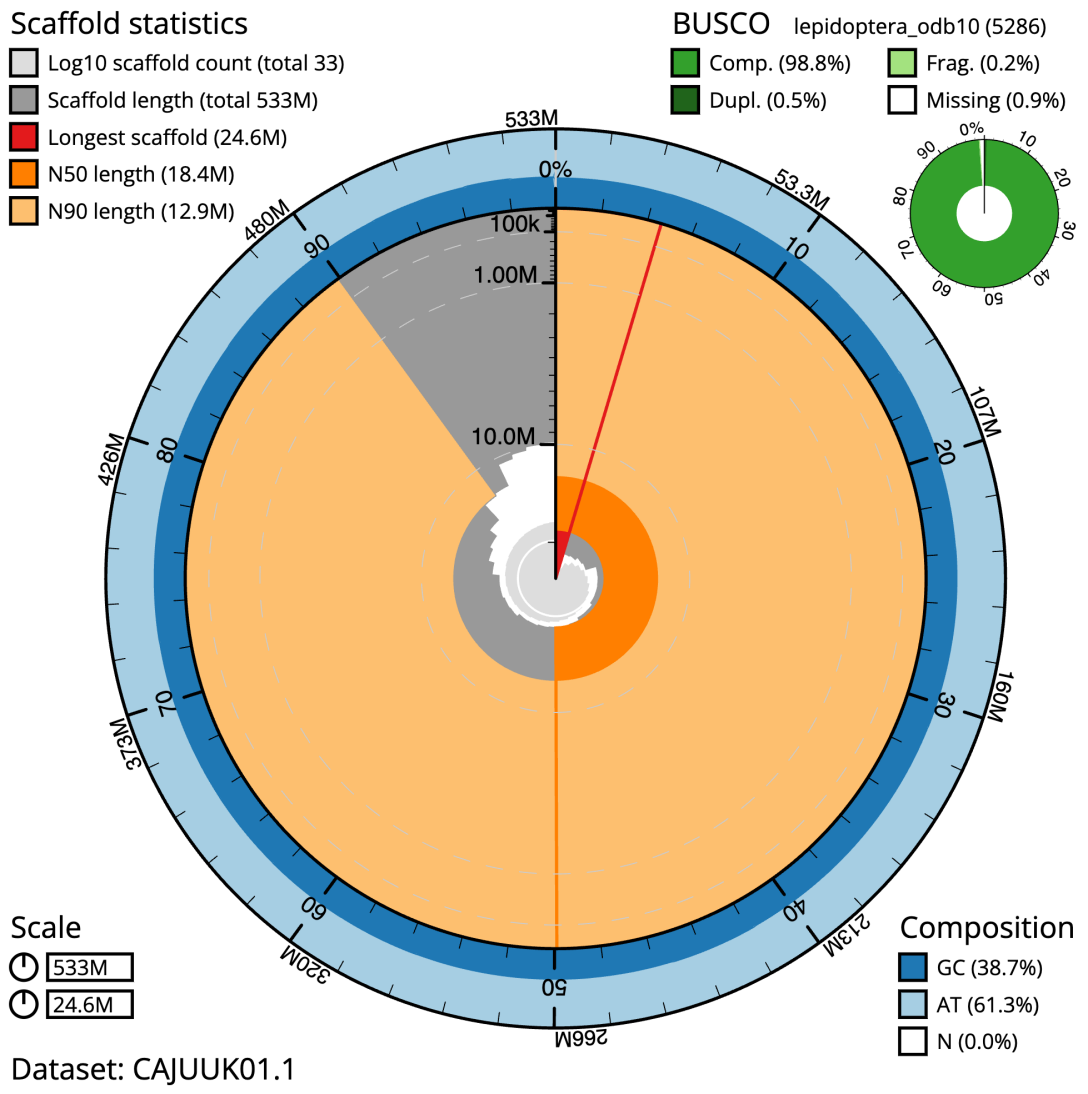
Genome assembly of *Noctua janthe*, ilNocJant1.1: metrics. The BlobToolKit Snailplot shows N50 metrics and BUSCO gene completeness. The main plot is divided into 1,000 size-ordered bins around the circumference with each bin representing 0.1% of the 532,786,062 bp assembly. The distribution of scaffold lengths is shown in dark grey with the plot radius scaled to the longest scaffold present in the assembly (24,567,908 bp, shown in red). Orange and pale-orange arcs show the N50 and N90 scaffold lengths (18,438,343 and 12,860,120 bp), respectively. The pale grey spiral shows the cumulative scaffold count on a log scale with white scale lines showing successive orders of magnitude. The blue and pale-blue area around the outside of the plot shows the distribution of GC, AT and N percentages in the same bins as the inner plot. A summary of complete, fragmented, duplicated and missing BUSCO genes in the lepidoptera_odb10 set is shown in the top right. An interactive version of this figure is available at https://blobtoolkit.genomehubs.org/view/ilNocJant1.1/dataset/CAJUUK01.1/snail.

The final assembly has a total length of 532.8 Mb in 32 sequence scaffolds with a scaffold N50 of 18.4 Mb ( Table 1). Most (99.99%) of the assembly sequence was assigned to 31 chromosomal-level scaffolds, representing 30 autosomes and the Z sex chromosome. Chromosome-scale scaffolds confirmed by the Hi-C data are named in order of size ( Figure 2– Figure 5; Table 2). While not fully phased, the assembly deposited is of one haplotype. Contigs corresponding to the second haplotype have also been deposited. The mitochondrial genome was also assembled and can be found as a contig within the multifasta file of the genome submission.

**Table 1.** Genome data for *Noctua janthe*, ilNocJant1.1.

Project accession data		
Assembly identifier	ilNocJant1.1	
Species	*Noctua janthe*	
Specimen	ilNocJant1	
NCBI taxonomy ID	987995	
BioProject	PRJEB45125	
BioSample ID	SAMEA7701537	
Isolate information	ilNocJant1	
Assembly metrics *		*Benchmark*
Consensus quality (QV)	61.4	*≥ 50*
*k*-mer completeness	100%	*≥ 95%*
BUSCO **	C:98.8%[S:98.3%,D:0.5%], F:0.2%,M:0.9%,n:5,286	*C ≥ 95%*
Percentage of assembly mapped to chromosomes	99.99%	*≥ 95%*
Sex chromosomes	Z chromosome	*localised homologous * *pairs*
Organelles	Mitochondrial genome assembled	*complete single alleles*
Raw data accessions		
PacificBiosciences SEQUEL II	ERR6436377	
10X Genomics Illumina	ERR6054797–ERR6054800	
Hi-C Illumina	ERR6054796	
Genome assembly		
Assembly accession	GCA_910589295.1	
*Accession of alternate* * haplotype*	GCA_910589505.1	
Span (Mb)	532.8	
Number of contigs	40	
Contig N50 length (Mb)	18.4	
Number of scaffolds	32	
Scaffold N50 length (Mb)	18.4	
Longest scaffold (Mb)	24.6	
Genome annotation		
Number of protein-coding genes	17,653	
Number of gene transcripts	17,848	

* Assembly metric benchmarks are adapted from column VGP-2020 of “Table 1: Proposed standards and metrics for defining genome assembly quality” from ( Rhie *et al.*, 2021). ** BUSCO scores based on the lepidoptera_odb10 BUSCO set using v5.3.2. C = complete [S = single copy, D = duplicated], F = fragmented, M = missing, n = number of orthologues in comparison. A full set of BUSCO scores is available at https://blobtoolkit.genomehubs.org/view/ilNocJant1.1/dataset/CAJUUK01.1/busco.

**Figure 3.**
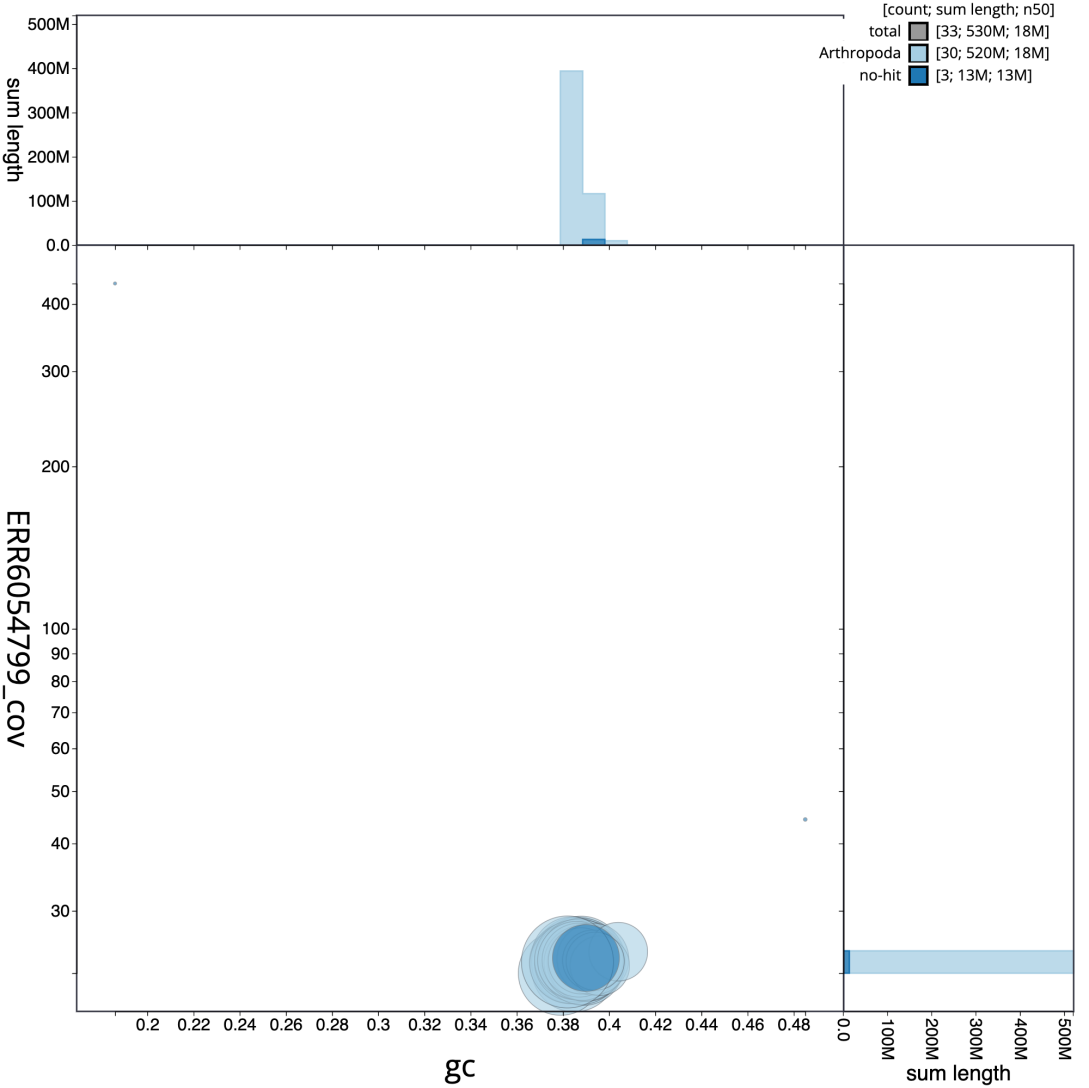
Genome assembly of *Noctua janthe*, ilNocJant1.1: BlobToolKit GC-coverage plot. Scaffolds are coloured by phylum. Circles are sized in proportion to scaffold length. Histograms show the distribution of scaffold length sum along each axis. An interactive version of this figure is available at https://blobtoolkit.genomehubs.org/view/ilNocJant1.1/dataset/CAJUUK01.1/blob.

**Figure 4.**
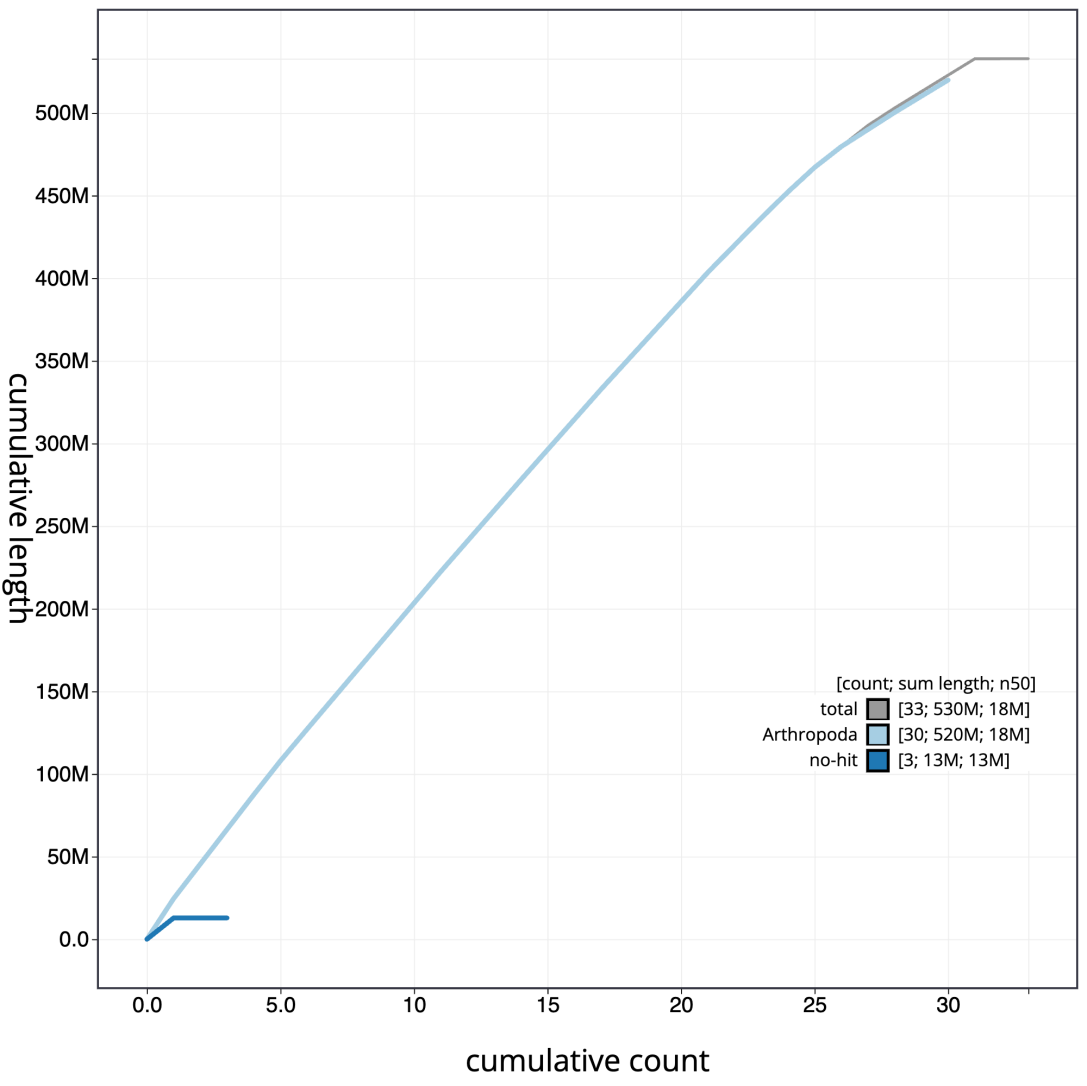
Genome assembly of *Noctua janthe*, ilNocJant1.1: BlobToolKit cumulative sequence plot. The grey line shows cumulative length for all scaffolds. Coloured lines show cumulative lengths of scaffolds assigned to each phylum using the buscogenes taxrule. An interactive version of this figure is available at https://blobtoolkit.genomehubs.org/view/ilNocJant1.1/dataset/CAJUUK01.1/cumulative.

**Figure 5.**
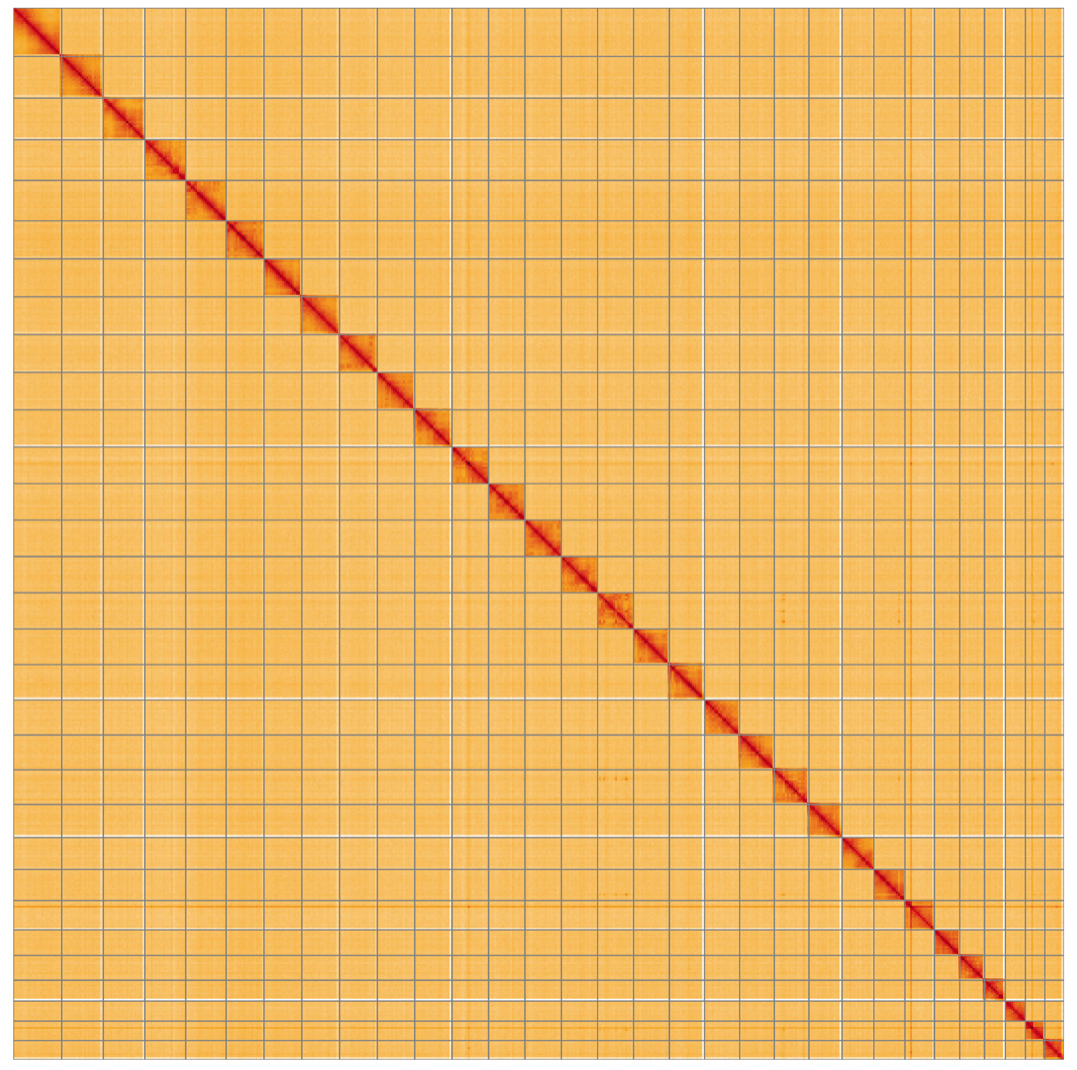
Genome assembly of Noctua janthe, ilNocJant1.1: Hi-C contact map of the ilNocJant1.1 assembly, visualised using HiGlass. Chromosomes are shown in order of size from left to right and top to bottom. An interactive version of this figure may be viewed at https://genome-note-higlass.tol.sanger.ac.uk/l/?d=G6TD3IGkSLCpXCG-r9Eogg.

**Table 2.** Chromosomal pseudomolecules in the genome assembly of *Noctua janthe*, ilNocJant1.

INSDC accession	Chromosome	Size (Mb)	GC%
OU342553.1	1	21.16	38.6
OU342554.1	2	21.03	38.5
OU342555.1	3	20.66	37.9
OU342556.1	4	20.5	38.8
OU342557.1	5	19.22	38.4
OU342558.1	6	19.2	38.8
OU342559.1	7	19.16	38.7
OU342560.1	8	19.15	38.4
OU342561.1	9	18.88	38.4
OU342562.1	10	18.86	38.3
OU342563.1	11	18.56	38.9
OU342564.1	12	18.49	38.3
OU342565.1	13	18.44	38.5
OU342566.1	14	18.36	38.5
OU342567.1	15	18.24	38.8
OU342568.1	16	18.19	39
OU342569.1	17	17.84	38.7
OU342570.1	18	17.76	38.6
OU342571.1	19	17.59	38.6
OU342572.1	20	17.56	39
OU342573.1	21	16.78	39
OU342574.1	22	16.14	38.8
OU342575.1	23	15.79	38.7
OU342576.1	24	14.85	39.3
OU342577.1	25	12.86	39
OU342578.1	26	12.62	38.8
OU342579.1	27	10.53	39.3
OU342580.1	28	10.17	39.6
OU342581.1	29	9.88	40.4
OU342582.1	30	9.71	39.4
OU342552.1	Z	24.57	38.2
OU342583.1	MT	0.02	18.9
-	unplaced	0.02	48.8

The estimated Quality Value (QV) of the final assembly is 61.4 with *k*-mer completeness of 100%, and the assembly has a BUSCO v5.3.2 completeness of 98.8% (single = 98.3%, duplicated = 0.5%), using the lepidoptera_odb10 reference set ( *n* = 5,286).

Metadata for specimens, spectral estimates, sequencing runs, contaminants and pre-curation assembly statistics can be found at https://links.tol.sanger.ac.uk/species/987995.

## Genome annotation report

The *Noctua janthe* GCA_910589295.1 genome assembly was annotated using the Ensembl rapid annotation pipeline ( Table 1; https://rapid.ensembl.org/Noctua_janthe_GCA_910589295.1/Info/Index). The resulting annotation includes 17,848 transcribed mRNAs from 17,653 protein-coding genes.

## Methods

### Sample acquisition and nucleic acid extraction

A male *Noctua janthe* (individual ilNocJant1; specimen Ox000676) was collected from was collected from Wytham Woods, Oxfordshire (biological vice-county Berkshire), UK (latitude 51.77, longitude –1.34) on 20 July 2020. The specimen was taken from woodland habitat by Douglas Boyes (University of Oxford) using a light trap. The specimen was identified by the collector and then snap-frozen on dry ice.

DNA was extracted at the Tree of Life laboratory, Wellcome Sanger Institute (WSI). The ilNocJant1 sample was weighed and dissected on dry ice with head and thorax tissue set aside for Hi-C sequencing. Abdomen tissue was cryogenically disrupted to a fine powder using a Covaris cryoPREP Automated Dry Pulveriser, receiving multiple impacts. High molecular weight (HMW) DNA was extracted using the Qiagen MagAttract HMW DNA extraction kit. Low molecular weight DNA was removed from a 20 ng aliquot of extracted DNA using the 0.8X AMpure XP purification kit prior to 10X Chromium sequencing; a minimum of 50 ng DNA was submitted for 10X sequencing. HMW DNA was sheared into an average fragment size of 12–20 kb in a Megaruptor 3 system with speed setting 30. Sheared DNA was purified by solid-phase reversible immobilisation using AMPure PB beads with a 1.8X ratio of beads to sample to remove the shorter fragments and concentrate the DNA sample. The concentration of the sheared and purified DNA was assessed using a Nanodrop spectrophotometer and Qubit Fluorometer and Qubit dsDNA High Sensitivity Assay kit. Fragment size distribution was evaluated by running the sample on the FemtoPulse system.

### Sequencing

Pacific Biosciences HiFi circular consensus and 10X Genomics read cloud DNA sequencing libraries were constructed according to the manufacturers’ instructions. DNA sequencing was performed by the Scientific Operations core at the WSI on Pacific Biosciences SEQUEL II (HiFi) and Illumina NovaSeq 6000 (10X) instruments. Hi-C data were also generated from head and thorax tissue of ilNocJant1 using the Arima2 kit and sequenced on the Illumina NovaSeq 6000 instrument.

### Genome assembly, curation and evaluation

Assembly was carried out with Hifiasm ( Cheng *et al.*, 2021) and haplotypic duplication was identified and removed with purge_dups ( Guan *et al.*, 2020). One round of polishing was performed by aligning 10X Genomics read data to the assembly with Long Ranger ALIGN, calling variants with FreeBayes ( Garrison & Marth, 2012). The assembly was then scaffolded with Hi-C data ( Rao *et al.*, 2014) using SALSA2 ( Ghurye *et al.*, 2019). The assembly was checked for contamination and corrected using the gEVAL system ( Chow *et al.*, 2016) as described previously ( Howe *et al.*, 2021). Manual curation was performed using gEVAL, HiGlass ( Kerpedjiev *et al.*, 2018) and Pretext ( Harry, 2022). The mitochondrial genome was assembled using MitoHiFi ( Uliano-Silva *et al.*, 2022), which runs MitoFinder ( Allio *et al.*, 2020) or MITOS ( Bernt *et al.*, 2013) and uses these annotations to select the final mitochondrial contig and to ensure the general quality of the sequence. To evaluate the assembly, MerquryFK was used to estimate consensus quality (QV) scores and *k*-mer completeness ( Rhie *et al.*, 2020). The genome was analysed within the BlobToolKit environment ( Challis *et al.*, 2020) and BUSCO scores ( Manni *et al.*, 2021; Simão *et al.*, 2015) were calculated. Table 3 contains a list of software tool versions and sources.

**Table 3.** Software tools: versions and sources.

Software tool	Version	Source
BlobToolKit	4.0.7	https://github.com/blobtoolkit/ blobtoolkit
BUSCO	5.3.2	https://gitlab.com/ezlab/busco
FreeBayes	1.3.1-17- gaa2ace8	https://github.com/freebayes/ freebayes
gEVAL	N/A	https://geval.org.uk/
Hifiasm	0.14	https://github.com/chhylp123/ hifiasm
HiGlass	1.11.6	https://github.com/higlass/higlass
Long Ranger ALIGN	2.2.2	https://support.10xgenomics. com/genome-exome/software/ pipelines/latest/advanced/other- pipelines
Merqury	MerquryFK	https://github.com/thegenemyers/ MERQURY.FK
MitoHiFi	2	https://github.com/marcelauliano/ MitoHiFi
PretextView	0.2	https://github.com/wtsi-hpag/ PretextView
purge_dups	1.2.3	https://github.com/dfguan/purge_ dups
SALSA	2.2	https://github.com/salsa-rs/salsa

### Genome annotation

The BRAKER2 pipeline ( Brůna *et al.*, 2021) was used in the default protein mode to generate annotation for the *Noctua janthe* assembly (GCA_910589295.1). in Ensembl Rapid Release.

### Ethics and compliance issues

The materials that have contributed to this genome note have been supplied by a Darwin Tree of Life Partner. The submission of materials by a Darwin Tree of Life Partner is subject to the Darwin Tree of Life Project Sampling Code of Practice. By agreeing with and signing up to the Sampling Code of Practice, the Darwin Tree of Life Partner agrees they will meet the legal and ethical requirements and standards set out within this document in respect of all samples acquired for, and supplied to, the Darwin Tree of Life Project. All efforts are undertaken to minimise the suffering of animals used for sequencing. Each transfer of samples is further undertaken according to a Research Collaboration Agreement or Material Transfer Agreement entered into by the Darwin Tree of Life Partner, Genome Research Limited (operating as the Wellcome Sanger Institute), and in some circumstances other Darwin Tree of Life collaborators.

## Data Availability

European Nucleotide Archive: *Noctua janthe* (Lesser Broad-bordered Underwing). Accession number PRJEB45125; https://identifiers.org/ena.embl/PRJEB45125. ( Wellcome Sanger Institute, 2021) The genome sequence is released openly for reuse. The *Noctua janthe* genome sequencing initiative is part of the Darwin Tree of Life (DToL) project. All raw sequence data and the assembly have been deposited in INSDC databases. Raw data and assembly accession identifiers are reported in Table 1.

## Author information

Members of the University of Oxford and Wytham Woods Genome Acquisition Lab are listed here: https://doi.org/10.5281/zenodo.4789928.

Members of the Darwin Tree of Life Barcoding collective are listed here: https://doi.org/10.5281/zenodo.4893703.

Members of the Wellcome Sanger Institute Tree of Life programme are listed here: https://doi.org/10.5281/zenodo.4783585.

Members of Wellcome Sanger Institute Scientific Operations: DNA Pipelines collective are listed here: https://doi.org/10.5281/zenodo.4790455.

Members of the Tree of Life Core Informatics collective are listed here: https://doi.org/10.5281/zenodo.5013541.

Members of the Darwin Tree of Life Consortium are listed here: https://doi.org/10.5281/zenodo.4783558.
