# Supplementary material for: The genome sequence of the Lesser Broad-bordered Yellow Underwing, Noctua janthe (Borkhausen, 1792)
Source: Wellcome Open Res. 2023 Apr 27;8:189. [Version 1] doi: 10.12688/wellcomeopenres.19412.1 (PMC10570690; doi:10.12688/wellcomeopenres.19412.1)
Supplement: Peer review history

# Reviewer report: Reviewer response for version 1

**DOI:** 10.21956/wellcomeopenres.21505.r67780

**Published:** 2024-03-01

**Authors:** Shota Nomura

**Affiliations:** 1 Division of Evolutionary Developmental Biology, National Institute for Basic Biology, Okazaki, Japan

This study performed the chromosome-level genome assembly of the Lesser Broad-bordered Yellow Underwing, *Noctua janthe*. Authors assembled the contigs using PacBio single-molecule HiFi long reads and 10X Genomics read clouds data and scaffolded them using Hi-C data. As a result, the authors obtained 32 scaffolds with 18.4 Mb of N50 and 98.8% of BUSCO score. Among the assembly, 99.99% were assigned to 30 autosomes and the Z sex chromosomes, resulting in a chromosome-level genome assembly with very high completeness.

 In this manuscript, the methods of analyses were appropriate and well explained. However, I think that there are some problems, including the question of whether the insect used in this study is truly *N. janthe*. I note below some questions.

 1. The authors explain taxonomic discussion in *N. janthe* in the Background section. However, it was not explained whether Wytham Woods, Oxfordshire, where the insects were sampled in this study, overlaps in distribution with the closely related species, *N. janthina*. If this sampling site is overlapping in distribution of *N. janthina*, the individuals used in this study and the genome sequences may have been affected by gene flow from *N. janthina* and may contain the parts of sequences which are different from the true *N. janthe* genome sequence. Although it would be ideal to show that the distribution of *N. janthina* does not overlaps in the sampling sites and that there is no gene flow, it is necessary to describe the relationship between the distribution area of *N. janthina* and the sampling sites and then authors should discuss whether the genome sequences are affected by closely related species.

 2. How did authors distinguish Z sex chromosomes from autosomes? If authors obtained male and female reads, they can be distinguished based on differences in reads mapping between the sexes. Did the authors use such a method? In any case, it would be better to be explained the methods of distinguish them.

Are sufficient details of methods and materials provided to allow replication by others?

Partly

Is the rationale for creating the dataset(s) clearly described?

Yes

Are the datasets clearly presented in a useable and accessible format?

Yes

Are the protocols appropriate and is the work technically sound?

Yes

Reviewer Expertise:

Evolutionary ecology, genomics, RNA-seq, insect, Carabidae, speciation, sexual selection

I confirm that I have read this submission and believe that I have an appropriate level of expertise to confirm that it is of an acceptable scientific standard, however I have significant reservations, as outlined above.

# Reviewer report: Reviewer response for version 1

**DOI:** 10.21956/wellcomeopenres.21505.r67783

**Published:** 2023-10-12

**Authors:** Hermes E. Escalona

**Affiliations:** 1 Australian National Insect Collection, CSIRO, Canberra, Australia

This is another valuable contribution from DTOL to Lepidoptera genomes. The text is clearly written with an appropriated context and references. 

 The methodology is clear and detailed but may be some more information on the genome annotation will be handy. For example, which data was used as input for the BRAKER2 pipeline, since it looks like its transcriptome was not sequenced during this project?

Are sufficient details of methods and materials provided to allow replication by others?

Yes

Is the rationale for creating the dataset(s) clearly described?

Yes

Are the datasets clearly presented in a useable and accessible format?

Yes

Are the protocols appropriate and is the work technically sound?

Yes

Reviewer Expertise:

Entomology

I confirm that I have read this submission and believe that I have an appropriate level of expertise to confirm that it is of an acceptable scientific standard.

# Reviewer report: Reviewer response for version 1

**DOI:** 10.21956/wellcomeopenres.21505.r67784

**Published:** 2023-10-09

**Authors:** Fahad Alqahtani

**Affiliations:** 1 National Center for Agricultural Technology, National Center for Agricultural Technology, King Abdulaziz City for Science and Technology, Riyadh, Saudi Arabia

The manuscript titled "The genome sequence of the Lesser Broad-bordered Yellow Underwing, Noctua janthe (Borkhausen, 1792)", successfully reconstructed the genome sequence of the Lesser Broad-bordered Yellow Underwing using three sequencing technologies: PacBio HiFi long reads, 10X Genomics, and Hi-C. The authors achieved an average coverage of 48X and 79X for PacBio HiFi long reads and 10X Genomics reads, respectively.

 The manuscript is well organized, with a clear and logical flow throughout the content, demonstrating a significant contribution to the field of genomics. The methodology section is detailed and thoroughly explained.

 To assemble the PacBio reads, the authors utilized Hifiasm and polished the PacBio contigs with 10X Genomics reads using the longranger align tool. Additionally, they performed scaffolding with Hi-C data using SALSA2. The final genome assembly comprised 32 sequence scaffolds, representing 30 chromosomes and the sex chromosome (Z), totaling 532,800,000 base pairs. The assembly's quality was assessed using BUSCO, yielding high scores.

 However, there are a few minor issues that need addressing. In the method section (Sample acquisition and nucleic acid extraction), there is a repetition of "was collected from was collected from." Additionally, in the method section (Genome assembly, curation, and evaluation), the authors mentioned MitoHiFi runs either MitoFinder or MITOS. It would be helpful to specify which of these tools, or both, were used. If MitoHiFi/Mitofinder was used for guiding the annotation of the mitochondrial genome, mentioning the closely related species used would be beneficial. Furthermore, in the method section (Genome assembly, curation, and evaluation), the term "Pretext" should be corrected to "PretextView."

Are sufficient details of methods and materials provided to allow replication by others?

Yes

Is the rationale for creating the dataset(s) clearly described?

Yes

Are the datasets clearly presented in a useable and accessible format?

Yes

Are the protocols appropriate and is the work technically sound?

Yes

Reviewer Expertise:

Bioinformatics

I confirm that I have read this submission and believe that I have an appropriate level of expertise to confirm that it is of an acceptable scientific standard, however I have significant reservations, as outlined above.

# Reviewer report: Reviewer response for version 1

**DOI:** 10.21956/wellcomeopenres.21505.r65333

**Published:** 2023-09-06

**Authors:** Boyd A Mori

**Affiliations:** 1 Department of Agricultural, Food and Nutritional Science, University of Alberta, Edmonton, AB, Canada

This data note presents the first genome assembly and annotation of the lesser broad-bordered yellow underwing, *Noctua janthe*. Given the discussion on whether or not *N. janthe* is a distinct species from *N. janthina*, this genome may help future taxonomists resolve the matter. 

 Standard metrics confirm a high quality assembly. 

 As noted with other Tree of Life projects, the methods are sparse, but adequate. A supplementary file of parameter settings for software use would be of benefit to others conducting similar assemblies. 

 The genome was annotated with Braker2 without transcriptome data/RNAseq reads for support. Future work, could support these annotations with transcript data. 

 In the current version, there is repetition in the methodology section of "was collected from was collected from"

Are sufficient details of methods and materials provided to allow replication by others?

Partly

Is the rationale for creating the dataset(s) clearly described?

Yes

Are the datasets clearly presented in a useable and accessible format?

Yes

Are the protocols appropriate and is the work technically sound?

Yes

Reviewer Expertise:

Agricultural entomology, insect genomics

I confirm that I have read this submission and believe that I have an appropriate level of expertise to confirm that it is of an acceptable scientific standard.
